# Supplementary material for: The genome sequence of the Twenty-plume Moth, Alucita hexadactyla Linnaeus, 1758
Source: Wellcome Open Res. 2025 Feb 13;10:70. [Version 1] doi: 10.12688/wellcomeopenres.23736.1 (PMC11880763; doi:10.12688/wellcomeopenres.23736.1)
Supplement: Peer review history

# Reviewer report: Reviewer response for version 1

**DOI:** 10.21956/wellcomeopenres.26184.r118753

**Published:** 2025-03-13

**Authors:** Toni De-Dios

**Affiliations:** 1 Institute of Genomics, University of Tartu, Tartu, Estonia

In the present article, Crowley and colleagues report a new genome assembly for *Alucita hexadactyla*, a common moth that represents the only member of the Alucitidae family in the UK. The resultant assembled genome is of high quality, with and average depth of 34X, with 99.75% of the sequences contained in the 30 assembled chromosomes (autosomes and sexual chromosomes), a quality value of 63.5%, combined *k-mer *completeness of 99.02%, and BUSCO of 98.1%. The protocols used to achieve these results are state-of-the-art, and they are explained in the detail in the methods sections. All software used is properly detailed and cited. The assembled genome is publicly available at ENA, and the raw data at INSDC.

 The results of this piece of work are clear and solid, with this high-quality assembly setting a new precedent for the study of the population genomics of the moth *Alucita hexadactyla*.

Are sufficient details of methods and materials provided to allow replication by others?

Yes

Is the rationale for creating the dataset(s) clearly described?

Yes

Are the datasets clearly presented in a useable and accessible format?

Yes

Are the protocols appropriate and is the work technically sound?

Yes

Reviewer Expertise:

Ancient DNA, population genetics, metagenomics, phylogenetics.

I confirm that I have read this submission and believe that I have an appropriate level of expertise to confirm that it is of an acceptable scientific standard.

# Reviewer report: Reviewer response for version 1

**DOI:** 10.21956/wellcomeopenres.26184.r118751

**Published:** 2025-03-04

**Authors:** Annabel Whibley

**Affiliations:** 1 The University of Auckland, Auckland, New Zealand; 2 Grapevine Improvement, Bragato Research Institute, Lincoln, Canterbury, New Zealand

Crowley and colleagues report the genome assembly of the twenty plume moth, *Alucita hexadactyla* , a reference that serves as an important “first” for the Alucitoidea superfamily. The analysis follows standardised Darwin Tree of Life sampling, verification, sequencing and reporting protocols. As usual, these are comprehensive, clearly structured and using current best-practice tools. That said, this is the first data note that I have reviewed that decomposes kmer completeness into primary and alternative haplotypes, and more information on methods has been incorporated, such as the reporting of kmer lengths used for analyses,  and I welcome these changes to the template. A very high quality reference is reported, starting with >100x HiFi data and Illumina HiC data.  RNAseq data was obtained but an annotation has not been reported. Public data accession links are active.

Are sufficient details of methods and materials provided to allow replication by others?

Yes

Is the rationale for creating the dataset(s) clearly described?

Yes

Are the datasets clearly presented in a useable and accessible format?

Yes

Are the protocols appropriate and is the work technically sound?

Yes

Reviewer Expertise:

Genomics Bioinformatics

I confirm that I have read this submission and believe that I have an appropriate level of expertise to confirm that it is of an acceptable scientific standard.

# Reviewer report: Reviewer response for version 1

**DOI:** 10.21956/wellcomeopenres.26184.r118756

**Published:** 2025-03-01

**Authors:** Daniel Berner

**Affiliations:** 1 University of Basel, Basel, Switzerland

This paper describes the assembly and annotation of a reference genome in chromosome quality for the Twenty-plume Moth *Alucita hexadactyla*. Based on samples from the UK, the work involves PacBio HiFi long-read sequencing and Hi-C link information to assemble 30 chromosomes, including the Z (the assembly is based on a male specimen lacking the W chromosome). The overall strategy follows the Darwin Tree of Life pipeline. Based on different completeness and contiguity checks, the genome can be considered of top quality. In addition, whole-organism RNA sequencing is performed for a de novo annotation, although this annotation is not included in this paper.

 The samples, wet laboratory procedures and analytical pipelines are comprehensively described, and accession numbers for the output are provided, allowing the community to use this genome as a resource. I do not have any comments to the authors apart from my recommendation to add the annotation to this paper if possible, to make this genomic resource more complete. Since the RNA seq data are already generated, I feel this would not be too much extra work.

Are sufficient details of methods and materials provided to allow replication by others?

Yes

Is the rationale for creating the dataset(s) clearly described?

Yes

Are the datasets clearly presented in a useable and accessible format?

Yes

Are the protocols appropriate and is the work technically sound?

Yes

Reviewer Expertise:

Evolutionary ecology; Population genomics; Speciation

I confirm that I have read this submission and believe that I have an appropriate level of expertise to confirm that it is of an acceptable scientific standard.

# Reviewer report: Reviewer response for version 1

**DOI:** 10.21956/wellcomeopenres.26184.r118757

**Published:** 2025-02-19

**Authors:** Jesper Boman

**Affiliations:** 1 Uppsala University, Uppsala, Sweden

Crowley et al. presents a high-quality genome assembly of the twenty-plume moth ( *Alucita hexadactyla*). As the authors state, this assembly is useful for further evolutionary analysis of Alucitidae. Background is overall well-written. See below for some comments.

 1. First, I appreciate the thoroughness in barcoding the samples to make sure of species status.

 2. In the Background section you write " *Lonicera caprifolium, periclymenum, xylosteum*". Based on my knowledge it is more common to write: *Lonicera caprifolium, L. periclymenum, L. xylosteum.*

 3 You write: "The Alucitidae family is relatively small for a lepidopteran family, with roughly 180 species ( Watkins, 2005), but is still expanding." It would be more appropriate to write that more species in Alucitidae are being described rather than that it is expanding, which gives connotations of a biological process such as a diversfication rate larger than zero. The latter might be true but I think it is the former that the authors are trying to say.

 I also want to reiterate point 1), 2) and 4) from a previous peer review:

 Boman J. Peer Review Report For: The genome sequence of the Bright-line Brown-eye moth, Lacanobia oleracea Linnaeus, 1758 [version 1; peer review: 1 approved with reservations]. Wellcome Open Res 2024, 9:515 (https://doi.org/10.21956/wellcomeopenres.25228.r98875)

 They are valid for this report as well and addressing them would improve clarity and reproducibility.

 Best regards,

 Jesper

Are sufficient details of methods and materials provided to allow replication by others?

Yes

Is the rationale for creating the dataset(s) clearly described?

Yes

Are the datasets clearly presented in a useable and accessible format?

Partly

Are the protocols appropriate and is the work technically sound?

Partly

Reviewer Expertise:

Speciation genomics, Population genomics, Butterflies, Gene regulation, Transposons, Genomics

I confirm that I have read this submission and believe that I have an appropriate level of expertise to confirm that it is of an acceptable scientific standard, however I have significant reservations, as outlined above.
